# Cytokine Level Changes in Schizophrenia Patients with and without Metabolic Syndrome Treated with Atypical Antipsychotics

**DOI:** 10.3390/ph14050446

**Published:** 2021-05-09

**Authors:** Anastasiia S. Boiko, Irina A. Mednova, Elena G. Kornetova, Valeria I. Gerasimova, Alexander N. Kornetov, Anton J. M. Loonen, Nikolay A. Bokhan, Svetlana A. Ivanova

**Affiliations:** 1 Mental Health Research Institute, Tomsk National Research Medical Center of the Russian Academy of Sciences, Aleutskaya Str., 4, 634014 Tomsk, Russia; anastasya-iv@yandex.ru (A.S.B.); irinka145@yandex.ru (I.A.M.); havssaltvg@gmail.com (V.I.G.); bna909@gmail.com (N.A.B.); ivanovaniipz@gmail.com (S.A.I.); 2 University Hospital, Siberian State Medical University, Moskovsky Trakt, 2, 634050 Tomsk, Russia; 3 Fundamental Psychology and Behavioral Medicine Department, Siberian State Medical University, Moskovsky Trakt, 2, 634050 Tomsk, Russia; alkornetov@gmail.com; 4 PharmacoTherapy, -Epidemiology and -Economics, Groningen Research Institute of Pharmacy, University of Groningen, Antonius Deusinglaan 1, 9713AV Groningen, The Netherlands; a.j.m.loonen@rug.nl; 5 Psychiatry, Addictology and Psychotherapy Department, Siberian State Medical University, Moskovsky Trakt, 2, 634050 Tomsk, Russia

**Keywords:** schizophrenia, atypical antipsychotics, cytokine, metabolic syndrome

## Abstract

The present study aims at comparing the change in cytokine levels in schizophrenia patients treated with atypical antipsychotics, with or without metabolic syndrome (MetS). The study included 101 patients with schizophrenia, 38 with and 63 without MetS, who received risperidone, quetiapine, olanzapine or aripiprazole for six weeks. We analyzed the concentration of 21 cytokines in the serum patients. The treatment with atypical antipsychotics changed some proinflammatory cytokine levels. It led to increased IFN-α2 (*p* = 0.010), IL-1α (*p* = 0.024) and IL-7 (*p* = 0.017) levels in patients with MetS, whereas the same treatment led to decreased levels of IFN-γ (*p* = 0.011), IL-1β (*p* = 0.035), IL-12р40 (*p* = 0.011), IL-17A (*p* = 0.031), IL-6 (*p* = 0.043) and TNF-α (*p* = 0.012) in individuals without MetS. Our results demonstrated the effects of atypical antipsychotics on the immune–inflammatory parameters, depending on the metabolic disturbances in schizophrenia patients.

## 1. Introduction

Immunoinflammation plays an important role in the pathogenetic mechanisms of schizophrenia, as evidenced by the activation of microglia demonstrated in neuroimaging studies, which was followed by the formation of inflammatory mediators [1,2]. These modulators affect neighboring astrocytes and neurons, making a significant contribution to the homeostatic regulation of brain tissue [3,4]. In some patients, an acute psychotic episode is associated with mild systemic inflammation, which is reflected by increased concentrations of cytokines and other inflammatory markers in the peripheral blood. It is assumed that inflammation is not only a consequence of schizophrenia but may also be a risk factor for its development [5]. In addition, the immunomodulatory and anti-inflammatory effects of antipsychotics support the role of inflammation in schizophrenia [6,7,8]. However, the effects of individual psychotropic agents on the immune system and how this might contribute to their effectiveness largely remains unclear [9].

Various studies have tried to assess the effects of adding anti-inflammatory drugs (aspirin, celecoxib, minocycline, acetylcysteine, omega-3 fatty acids, cytokines and interferon (IFN)-γ) to antipsychotic therapy; however, most of these studies lack sufficient methodological rigidity [10,11]. Further developing this type of treatment of patients with schizophrenia requires not only research clarifying how the anti-inflammatory effects of these treatments affect the symptoms of schizophrenia themselves but, also, how they interact with the pharmacological effects of antipsychotics [12]. Besides, the unique mechanisms by which proinflammatory cytokines are involved in the etiopathology of schizophrenia should be investigated [13]. Exploring how systemic factors such as metabolic syndrome (MetS) are involved should also be taken into account.

MetS has a high prevalence in patients with schizophrenia due to predisposition, as well as due to the long-term use of antipsychotics [14]. The incidence of MetS in patients with schizophrenia taking antipsychotics varies from 28% [15] to 46% [16] and occurs already during the first psychotic episode, whereas its frequency subsequently depends on the duration of the disease [17].

There is evidence that patients with schizophrenia have a metabolic predisposition to diabetes that is exacerbated by obesity, leading to cardiovascular disease and other comorbidities. In turn, the influence of the inflammatory mediators on the brain not only contributes to the development of schizophrenia but, also, to the deterioration of health that accompanies this disease [18].

It was previously shown that the levels of inflammation markers are higher in patients with schizophrenia and MetS compared with the patients without it [19], and the levels of C-reactive protein, interleukin (IL)-6, leptin, IFN-γ and tumor necrosis factor (TNF)-α can serve as prognostic factors for the development of metabolic syndrome in patients with schizophrenia during treatment with antipsychotics [20,21].

Second-generation antipsychotics (SGA) are the most frequently used medications in the treatment of patients with schizophrenia in recent years. The spectrum of atypicality ranges from risperidone as the least atypical, followed by aripiprazole, amisulpride, lurasidone, ziprasidone, asenapine, quetiapine and olanzapine to clozapine, which is the most atypical of all antipsychotics [22]. They are more complex dopamine D2 receptor antagonists and involve other receptor targets that regulate dopamine and other neurotransmitters. Accordingly, atypical antipsychotics cause fewer movement side effects that are associated with a strong blockade of D2 receptors [22].

It is assumed that antipsychotic treatment changes the patterns of gene expression in adipocytes and leads them to an inflammatory state, which is related to metabolic disorders in patients [23]. Thus, the ex vivo stimulation of primary mononuclear cells of peripheral blood from healthy donors with both olanzapine and aripiprazole reduced the mRNA levels of IL-1β, IL-6 and TNF-α and led to a decrease in the concentrations of the IL-6 and TNF-α proteins. The multiplex approach revealed the additional suppression of IL-2 and secretion of the macrophage inflammatory protein MIP-1β and interferon gamma-induced protein 10 (IP-10). Similarly, the stimulation of the transformed human monocytic leukemia cell line THP-1 with olanzapine and aripiprazole resulted in a significant decrease in the expression and secretion of IL-1β and TNF-α [24]. Schizophrenia patients with and without concomitant MetS treated with olanzapine or clozapine had significantly higher plasma IL-6, IL-10 and TNF-α levels compared to the normal controls, but markedly lower plasma silencing information regulator 2-related enzyme 1 (SIRT1) levels and higher plasma IL-6 levels were observed in patients with MetS compared to patients without MetS [25].

In another study, after 24 weeks of treatment with paliperidone, a significant increase in the waist circumference was found, which positively correlated with a change in the number of leukocytes [26]. Additionally, not only the long-term antipsychotic treatment of patients with schizophrenia leads to metabolic disorders; the use of atypical antipsychotics even for 6 weeks of therapy in patients without MetS contributes to a significant increase in visceral obesity, an increase in glucose levels and dyslipidemia [27], and after 6 months of treatment, the percentage of patients with schizophrenia and MetS increases from 5% to 27% [28].

The present study aims at comparing the change in the cytokine levels in schizophrenia patients treated with atypical antipsychotics, with or without MetS. We hypothesized that atypical antipsychotics have different effects on the serum cytokine levels in schizophrenic patients with and without MetS.

## 2. Results and Discussion

### 2.1. Baseline Characteristics of Study Participants

The demographics and disease characteristics, as well as the antipsychotic therapy of the study population, are summarized in Table 1. A total of 101 patients with schizophrenia who received SGA were enrolled in the study: 38 of them (37.6%) had MetS and 63 (62.4%) did not. Most patients were taking risperidone (43, 42.6%), quetiapine (22, 21.8%), olanzapine (14, 13.9%) or aripiprazole (11, 10.9%) as their baseline antipsychotic therapy. In the remaining cases, the patients received other SGA, but none received clozapine.

### 2.2. Cytokine Levels Depending on the Presence of MetS in Patients with Schizophrenia Receiving SGA

The treatment with atypical antipsychotics led to increased IFN-α2 (*p* = 0.010), IL-1α (*p* = 0.024) and IL-7 (*p* = 0.017) levels in patients with MetS, whereas the same treatment led to decreased levels of IFN-γ (*p* = 0.011), IL-1β (*p* = 0.035), IL-12р40 (*p* = 0.011), IL-17A (*p* = 0.031), IL-6 (*p* = 0.043) and TNF-α (*p* = 0.012) in individuals without MetS (Table 2).

**Figure 1:**
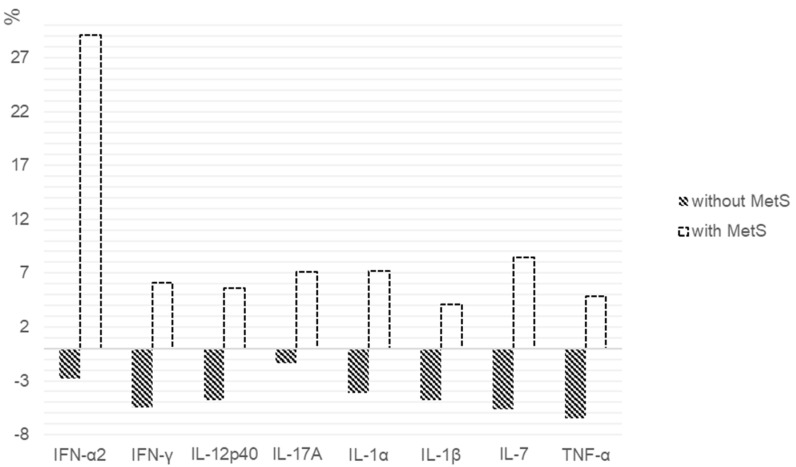
Percentage change in the cytokine levels after 6 weeks of SGA therapy in patients with and without MetS. We calculated the individual percentage changes in the indicator for each patient separately using the formula: ((V2 − V1)/V1) × 100, where V1 is the initial value before therapy, and V2 is the final value after therapy. The data in the figure are presented as the median of the changes in the indicators. The figure shows only the statistically significant differences according to the Mann–Whitney *U* test.

When assessing the percentage of changes in the cytokines levels between patients with MetS and without it, statistically significant differences in the IFN-α2 (*p* = 0.028), IFN-γ (*p* = 0.007), IL-12p40 (*p* = 0.007), IL-17A (*p* = 0.009), IL-1α (*p* = 0.011), IL-1β (*p* = 0.008), IL-7 (*p* = 0.004) and TNF-α (*p* = 0.031) level were found (Figure 1).

### 2.3. Cytokine Levels in Patients with Schizophrenia Depending on the Presence of MetS When Receiving Risperidone

The next stage of our study was to evaluate the effect of 6 weeks of risperidone treatment on the serum cytokine levels in patients with schizophrenia. A total 14 participants who take risperidone had a MetS, and 29 did not. In patients with MetS, 6 weeks of risperidone therapy led to increased IFN-α2 (*p* = 0.008), IL-1β (*p* = 0.041) and IL-7 (*p* = 0.046) levels. We did not find any changes in the cytokine levels in the individuals without MetS who received risperidone for 6 weeks (Table 3).

**Figure 2:**
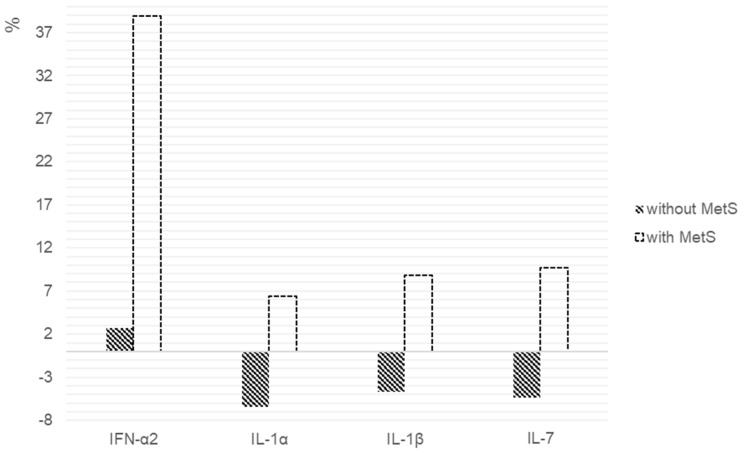
Percentage changes in cytokine levels after 6 weeks of risperidone therapy in patients with and without MetS. We calculated the individual percentage changes in the indicators for each patient separately using the formula: ((V2 − V1)/V1) × 100, where V1 is the initial value before therapy, and V2 is the final value after therapy. The data in the figure are presented as the median of the changes in the indicators. The figure shows only the statistically significant differences according to the Mann–Whitney *U* test.

The percentage changes in the cytokines levels, as shown in Figure 2. We found statistically significant differences in the IFN-α2 (*p* = 0.047), IL-1α (*p* = 0.017), IL-1β (*p* = 0.017) and IL-7 (*p* = 0.047) levels between the patients with and without MetS.

### 2.4. Cytokine Levels in Patients with Schizophrenia Depending on the Presence of MetS When Receiving Quetiapine or Olanzapine

Since quetiapine and olanzapine have approximately the same effect on the metabolic parameters [29], we pooled patients who received these drugs into one group to increase the sample size. In patients with MetS, 6 weeks of therapy led to an increased IFN-α2 (*p* = 0.050) level and, in patients without MetS, to decreased IL-6 (*p* = 0.041) and IL-12p40 (*p* = 0.050) (Table 4).

**Figure 3:**
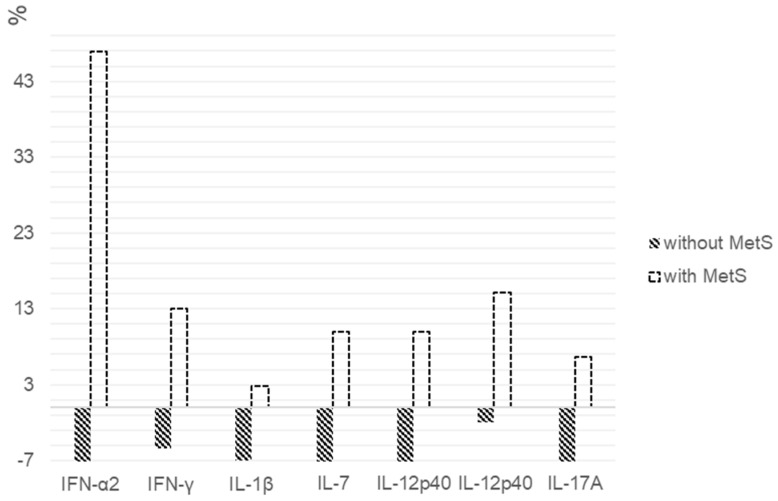
Percentage changes in cytokine levels after 6 weeks of quetiapine or olanzapine therapy in patients with and without MetS. We calculated the individual percentage changes in the indicators for each patient separately using the formula: ((V2 − V1)/V1) × 100, where V1 is the initial value before therapy, and V2 is the final value after therapy. The data in the figure are presented as the median of the changes in the indicators. The figure shows only the statistically significant differences according to the Mann–Whitney *U* test.

When assessing the percentages of the changes in cytokine levels between patients with MetS and without it, statistically significant differences in the IFN-α2 (*p* = 0.034), IFN-γ (*p* = 0.037), IL-12p40 (*p* = 0.011), IL-12p70 (*p* = 0.003), IL-17A (*p* = 0.002), IL-1β (*p* = 0.034) and IL-7 (*p* = 0.001) levels were found (Figure 3).

### 2.5. Cytokine Levels in Patients with Schizophrenia Depending on the Presence of MetS When Receiving Aripiprazole

All patients treated with aripiprazole had no MetS. We did not find any changes in the serum cytokines levels in the patients who received aripiprazole (Table 5).

## 3. Materials and Methods

The study protocol was approved by the Local Bioethics Committee of the Mental Health Research Institute, Tomsk, Russian Federation (#187, from 24 April 2018). One hundred and one patients with schizophrenia, according to the International Statistical Classification of Diseases and Related Health Problems, 10th Revision (ICD-10: F20), 18–55 years of age, were included in the study during 2018–2020, after having signed informed consent forms. We did not include schizophrenia patients with acute and chronic infectious, inflammatory and autoimmune diseases or patients who used psychoactive substances or took medications that could affect the metabolic or immunological parameters. Most patients received SGAs in anti-relapse and maintenance dosages before admission to the hospital. They were often nonadherent, and were hospitalized due to schizophrenia exacerbation. We divided the schizophrenia patients into two groups, depending on the presence of MetS: 63 of the patients met the criteria for MetS, according to the International Diabetes Federation (IDF, 2005). MetS is diagnosed when a patient has a central obesity (waist circumference more than 94 cm in men and more than 80 cm in women) and any two of the following four signs: (1) the concentration of triglycerides in the serum is higher than 1.7 mmol/L (150 mg/dL), or lipid-lowering therapy is carried out; (2) the concentration of high-density lipoproteins in the serum is below 1.03 mmol/L (40 mg/dL) in men and 1.29 mmol/L (50 mg/dL) in women; (3) the arterial blood pressure level is systolic above 130 mmHg or diastolic above 85 mmHg (or when using treatment for previously diagnosed hypertension); (4) the serum glucose concentration is greater than 5.6 mmol/L (100 mg/dL) (or previously diagnosed type 2 diabetes). Anthropometric and biochemical studies were carried out as previously described [30]. The observation period was 6 weeks, during which the patients were in the hospital. The severity of the schizophrenia symptoms was assessed with the Positive and Negative Syndrome Scale [31] at the beginning of the observation.

Blood for the biochemical analysis was taken from all subjects in the first days of hospitalization and, after a 6-week treatment with SGAs, after 12-h overnight fasting. Blood was centrifuged for 30 min at 2000× *g* at 4 °C to isolate the serum. Quantitative analyses of the cytokine concentrations in the serum were executed using the multi-analyte panel HCYTMAG-60K-PX41 by MILLIPLEX^®^ MAP (Merck, Darmstadt, Germany) on the multiplex analyzer MAGPIX (Luminex, Austin, TX, USA).

Statistical analysis of the data was performed using SPSS software (version 20) for Windows. We used several methods of group comparisons, such as the Shapiro–Wilk test, the Mann–Whitney *U* test, the Wilcoxon’s test and the chi-square test. Statistically significant differences were considered *p*-values less than 0.05.

## 4. Discussion

Metabolic disorders are frequently observed in patients with schizophrenia; they debuted from its early onset and showed an increase relative to its duration [17]. The differences in the schizophrenia duration between the groups obtained in this study agree with that observation. Additionally, patients with MetS had a longer duration of antipsychotic therapy. It is known that a long duration of illness and longer exposure to antipsychotics can be one of the risk factors for the development of MetS in patients with schizophrenia [32]. We found that patients with MetS had a significantly higher age and duration of schizophrenia in comparison with subjects without MetS. The results may be explained by the fact that the older the age of patients with schizophrenia, the more vulnerable they are to MetS development.

To the best of our knowledge, this is the first study that investigated the changes of the cytokine profile during SGA treatments in schizophrenia patients with the absence or presence of MetS. We studied the changes in the levels of 21 proinflammatory or anti-inflammatory cytokines.

We found that 6 weeks of therapy with SGAs did not affect the anti-inflammatory cytokines such as the IL-1 receptor antagonist (IL-1RA), IL-4 and IL-10, regardless of the presence or absence of MetS. The absence of SGA effects on the anti-inflammatory cytokines was demonstrated in a meta-analysis by Tourjman et al. (2013) [33]. In a 6-week study, taking SGA (risperidone or olanzapine) during the first episode of schizophrenia (FEP), patients with a normal BMI resulted in a decrease in IL-1RA and IL-10 [34]. Noto et al. [35] demonstrated a decrease of IL-4 and IL-10 in FEP patients who received risperidone over 10 weeks. In some in vitro and in vivo studies, a change in anti-inflammatory cytokines under the influence of antipsychotics was demonstrated [36,37]. It is tempting to speculate that the absence of the effect of antipsychotics on anti-inflammatory cytokines in our study had something to do with the adipose tissue protein–adiponectin. Adiponectin can enhance the production of anti-inflammatory mediators [38]. However, a long-term treatment with SGAs led to decreased adiponectin level [39], which, accordingly, canceled its ability to affect anti-inflammatory cytokines. In our study, patients with schizophrenia received antipsychotics for a long time and, as shown in previous research, had low adiponectin levels [30].

One meta-analysis demonstrated the anti-inflammatory effects of antipsychotics in patients with schizophrenia, but the authors noted that most studies did not control for potential confounding factors, such as BMI and smoking [6]. We found, in our study, that 6 weeks of antipsychotic treatment led to an increase in some proinflammatory cytokines such as the IFN-α2, IL-1α and IL-7 levels in patients with MetS and to decreased IFN-γ, IL-1β, IL-12р40, IL-6 and TNF-α in patients without MetS. We assume that our findings are associated with a deterioration in the metabolic status, which, in turn, is closely related to the chronic disease [20,21,40]. Changes in the serum levels of proinflammatory cytokines during SGA treatment may reflect a direct anti-inflammatory effect of the antipsychotics and an increased production of proinflammatory cytokines, depending on the metabolic parameters of the patients.

We found a decrease in the IL-17A level during 6 weeks of SGA therapy in schizophrenia patients without MetS. We want to emphasize that IL-17A is produced by T-helper 17 lymphocytes (Th-17 cells) and is mainly associated with autoimmune diseases [41]. No difference in the IL-17A levels between healthy individuals and patients with FEP was demonstrated in a recent meta-analysis [42]. There is a limited number of studies on the effect of antipsychotics on Th-17 cells. In particular, Dimitrov et al. 2013 [43] showed a decrease of the IL-17A levels in a patient with a long-standing diagnosis of chronic schizophrenia taking antipsychotics. The addition of antipsychotics to the whole blood of healthy women led to an increase in the IL-17 levels [44]. Thus, antipsychotics might have a direct effect on the level of IL-17; however, the mechanisms of the revealed changes require further study.

Risperidone therapy led to an increase in IFN-α2, IL-1β and IL-7 in schizophrenia patients with MetS. In turn, no changes in the cytokine levels in patients without MetS were found. We obtained similar results for the changes in the cytokine levels in patients treated with quetiapine and olanzapine. These findings are generally consistent with the study of Song et al. [40]. They showed that the short-term risperidone treatment of drug-naïve schizophrenia patients was associated with an initial anti-inflammatory effect that was reduced after 6 months of treatment, potentially due to its weight gain side effect. A decrease of the proinflammatory cytokines in drug-naïve FEP patients who received risperidone therapy for 10 weeks was also found in another study [35]. An increase in the white blood cell count associated with a raise in the waist volume was found after 24 weeks of treatment paliperidone—a major active metabolite of risperidone [26].

We were unable to confirm the data received by Sobiś et al. [45] about the anti-inflammatory effects of aripiprazole in patients with chronic schizophrenia. Aripiprazole is associated with a minimal effect on the weight gain, glucose level and lipid metabolism [46]. We did not find any effect of aripiprazole on the serum cytokine levels, but we only studied 11 patients.

In conclusion, we suggest that the anti-inflammatory effect of antipsychotics may worsen in the presence of chronic persistent inflammation caused by metabolic syndrome; however, further in-depth studies are needed to elucidate the mechanisms of these phenomena. In general, the results of our study once again emphasized the need for the secure treatment of patients concerning the possible adverse effects of antipsychotic therapy. Ultimately, we are not treating only schizophrenia but that the treatment affects the patient as a whole, and by preventing MetS, we can avoid the development of other serious disorders, including diabetes and cardiovascular diseases.

Our study is limited by the inability to fully evaluate the long-term therapy that patients received before their current hospitalization due to a lack of relevant information. On the other hand, the study represents a typical clinical situation where patients are hospitalized who already received long-term antipsychotic therapy. We want to emphasize that their metabolic status requires special attention, taking into account the pharmacological profiles of the antipsychotics used.

## Figures and Tables

**Table 1:** Sociodemographic, clinical and pharmacological characteristics of patients with schizophrenia, depending on the presence of MetS.

Indicators	Patients with MetS (n = 38)	Patients without MetS (n = 63)	*p*-Value
Age, Me (Q1; Q3) years	35 (30; 47)	30 (25; 36)	0.002 *
Gender (Male, n (%)/Female, n (%))	23 (60.5%)/15 (39.5%)	32 (50.8%)/31 (49.2%)	0.341
Schizophrenia onset age, Me [Q1; Q3] years	22 (19;27)	23.5 (19;29)	0.717
Duration of disease, Me [Q1; Q3] years	16 (5; 22)	6 (3; 11)	<0.001 *
PANSS positive symptoms score	19 (16; 23)	19 (14; 22)	0.405
PANSS negative symptoms score	25.5 (20; 30)	25 (21; 28)	0.629
PANSS general psychopathology symptoms score	51.5 (41; 54)	52 (43; 56)	0.735
PANSS total score	100.5 (85; 108)	96 (86; 104)	0.469
Antipsychotic therapy duration, years	5.5 (2; 16)	3 (0.2; 5)	0.002 *
Total antipsychotic dose, CPZeq	389.95 (200; 600)	360 (233; 609.75)	0.934
Smoking	23 (60.5%)	37 (58.7%)	0.237

Me (Q1; Q3)—median (lower quartile; upper quartile). MetS—metabolic syndrome. PANSS—positive and negative syndrome scale. CPZeq—Chlorpromazine equivalent. * *p* < 0.05—statistically significant difference. Comparisons between groups were performed using the chi-square test for gender and the Mann–Whitney *U* test for the other indicators.

**Table 2:** Serum concentrations of the cytokines (pg/mL) in patients with and without MetS during 6 weeks of SGA therapy, Me (Q1; Q3).

Indicators	Patients with MetS (n = 38)			Patients without MetS (n = 63)		
	Before	After	*p*-Value	Before	After	*p*-Value
IFN-α2	18.07 (10.78; 26.01)	23.54 (14.82; 33.96)	0.010 *	18.59 (14.22; 28.18)	21.26 (15.38; 28.90)	0.975
IFN-γ	10.06 (8.33; 12.04)	11.13 (9.17; 13.80)	0.119	10.85 (8.93; 13.67)	10.12 (9.14; 11.76)	0.011 *
IL-1RA	43.68 (38.13; 58.71)	48.79 (38.25; 64.82)	0.713	48.99 (38.38; 80.99)	47.14 (38.13; 73.21)	0.291
IL-1α	53.51 (47.66; 60.34)	55.74 (52.36; 63.92)	0.024 *	56.95 (50.02; 65.88)	54.67 (46.51; 63.73)	0.112
IL-1β	2.48 (1.99; 2.90)	2.64 (2.17; 3.10)	0.090	2.49 (1.88; 3.02)	2.33 (1.70; 2.74)	0.035 *
IL-2	4.74 (4.26; 5.12)	4.97 (4.42; 5.82)	0.063	5.15 (4.42; 6.11)	5.29 (4.62; 5.92)	0.986
IL-3	3.32 (2.90; 3.68)	3.32 (3.07; 3.73)	0.447	3.28 (2.81; 4.00)	3.52 (2.94; 4.02)	0.517
IL-4	75.27 (69.78; 87.86)	80.11 (71.35; 101.36)	0.245	79.28 (70.03; 97.11)	83.80 (70.40; 93.69)	0.866
IL-5	2.08 (1.39; 2.47)	1.98 (1.48; 2.66)	0.361	2.11 (1.57; 2.59)	1.98 (1.72; 2.58)	0.464
IL-6	6.13 (4.53; 8.18)	5.44 (4.48; 6.95)	0.274	5.94 (4.40; 9.61)	5.16 (3.81; 7.39)	0.043 *
IL-7	10.45 (8.98; 11.75)	10.67 (9.62; 12.71)	0.017 *	10.24 (8.87; 12.19)	9.65 (8.49; 11.96)	0.118
IL-8	14.25 (9.21; 19.97)	12.55 (8.98; 28.01)	0.258	12.35 (8.70; 20.70)	11.54 (8.61; 17.71)	0.271
IL-9	3.73 (2.86; 4.10)	3.84 (2.72; 4.39)	0.845	3.52 (2.61; 4.36)	3.42 (2.25; 4.10)	0.279
IL-10	9.11 (7.52; 11.95)	8.92 (7.08; 10.40)	0.572	7.99 (6.41; 10.81)	7.75 (6.24; 9.83)	0.335
IL-12p40	44.52 (38.30; 52.44)	44.85 (39.66; 53.38)	0.182	42.75 (34.27; 51.76)	40.18 (28.34; 51.72)	0.011 *
IL-12p70	6.78 (6.09; 9.82)	7.28 (6.57; 8.74)	0.072	7.26 (6.05; 8.66)	6.99 (6.14; 8.59)	0.352
IL-13	13.75 (12.18; 16.55)	13.42 (12.18; 18.23)	0.334	13.52 (11.45; 16.10)	12.97 (10.42; 16.83)	0.994
IL-15	6.90 (5.69; 8.53)	7.04 (5.65; 8.21)	0.839	6.26 (5.12; 8.23)	6.17 (4.93; 7.62)	0.183
IL-17A	4.39 (3.74; 5.24)	4.66 (4.14; 5.62)	0.150	4.47 (3.75; 5.34)	4.39 (3.47; 5.24)	0.031 *
TNF-α	20.75 (15.26; 26.26)	22.34 (16.15; 26.59)	0.342	18.22 (11.62; 25.48)	16.33 (12.10; 20.52)	0.012 *
TNF-β	6.78 (5.51; 8.83)	6.67 (5.59; 9.05)	0.604	6.49 (2.89; 11.92)	6.49 (2.50; 9.36)	0.199

Me (Q1; Q3)—median (lower quartile; upper quartile). IFN—interferon; IL—interleukin; TNF—tumor necrosis factor. *—*p* < 0.05 for the Wilcoxon signed-rank test.

**Table 3:** Serum concentrations of the cytokines (pg/mL) in patients with and without MetS during 6 weeks of risperidone therapy, Me (Q1; Q3).

Indicators	Patients with MetS (n = 14)			Patients without MetS (n = 29)		
	Before	After	*p*-Value	Before	After	*p*-Value
IFN-α2	17.95 (8.29; 23.98)	23.49 (12.39; 35.17)	0.008 *	17.53 (13.36; 27.83)	20.11 (15.62; 27.22)	0.633
IFN-γ	10.53 (8.78; 12.05)	11.31 (9.00; 13.59)	0.433	10.77 (8.90; 13.67)	10.51 (9.52; 11.71)	0.191
IL-1RA	43.86 (39.07; 57.35)	43.68 (39.07; 59.90)	0.859	46.65 (39.09; 78.24)	45.48 (38.25; 69.43)	0.641
IL-1α	54.08 (49.43; 58.69)	58.85 (50.02; 70.66)	0.084	56.95 (50.58; 65.88)	54.67 (45.85; 62.57)	0.111
IL-1β	2.68 (2.28; 2.87)	2.79 (2.44; 3.28)	0.041 *	2.49 (1.98; 3.23)	2.33 (1.83; 2.91)	0.225
IL-2	4.85 (4.37; 5.12)	5.34 (4.61; 5.78)	0.346	4.91 (4.38; 5.50)	5.19 (4.49; 5.80)	0.905
IL-3	3.32 (2.94; 3.73)	3.45 (3.07; 3.83)	0.289	3.07 (2.77; 3.86)	3.52 (3.07; 3.79)	0.319
IL-4	75.27 (72.14; 83.85)	85.97 (68.19; 107.62)	0.152	81.48 (69.68; 96.15)	83.85 (69.70; 134.07)	0.733
IL-5	1.78 (1.34; 2.46)	1.81 (1.42; 2.62)	0.600	2.09 (1.57; 2.81)	1.96 (1.51; 2.79)	0.464
IL-6	6.67 (4.67; 8.18)	5.44 (4.48; 7.35)	0.363	6.67 (4.46; 9.55)	5.56 (3.99; 8.28)	0.202
IL-7	10.84 (9.62; 11.75)	11.18 (10.05; 13.80)	0.046 *	10.18 (9.18; 11.67)	9.69 (8.90; 12.14)	0.948
IL-8	14.87 (11.71; 20.69)	13.97 (10.89; 21.83)	0.807	12.04 (8.77; 18.99)	13.03 (9.41; 17.36)	0.456
IL-9	3.78 (3.23; 4.13)	4.06 (3.24; 4.51)	0.706	3.57 (2.33; 4.93)	3.57 (2.71; 5.25)	0.673
IL-10	9.00 (7.08; 12.63)	8.84 (7.29; 11.42)	0.889	8.00 (7.08; 10.84)	8.00 (6.60; 1.01)	0.568
IL-12p40	45.73 (41.05; 52.31)	46.50 (40.18; 53.74)	0.397	44.52 (36.01; 62.32)	42.10 (32.29; 59.95)	0.315
IL-12p70	6.83 (6.66; 7.39)	7.47 (6.84; 8.45)	0.221	7.26 (6.23; 8.76)	7.08 (6.21; 8.59)	0.737
IL-13	12.97 (11.78; 14.32)	12.70 (11.98; 14.13)	0.944	13.75 (11.53; 17.90)	13.75 (10.60; 21.16)	0.341
IL-15	7.81 (6.93; 9.43)	7.80 (5.76; 8.79)	0.209	6.35 (5.39; 8.50)	6.70 (5.02; 7.95)	0.548
IL-17A	4.61 (3.95; 5.24)	5.30 (4.31; 6.81)	0.221	4.61 (3.75; 5.55)	4.50 (3.87; 5.39)	0.755
TNF-α	20.75 (18.67; 26.76)	22.79 (20.71; 26.48)	0.347	18.33 (12.84; 25.36)	17.46 (12.87; 20.17)	0.111
TNF-β	6.90 (6.13; 7.70)	6.93 (5.74; 8.52)	0.279	7.46 (3.44; 19.90)	6.78 (2.34; 18.72)	0.336

IFN—interferon; IL—interleukin; TNF—tumor necrosis factor. *—*p* < 0.05 for the Wilcoxon signed-rank test.

**Table 4:** Serum concentrations of the cytokines (pg/mL) in patients with and without MetS during 6 weeks of quetiapine or olanzapine therapy, Me (Q1; Q3).

Indicators	Patients with MetS (n = 13)			Patients without MetS (n = 23)		
	Before	After	*p*-Value	Before	After	*p*-Value
IFN-α2	17.50 (8.78; 27.07)	23.59 (13.54; 36.84)	0.050 *	18.87 (15.78; 27.67)	20.88 (15.38; 28.89)	0.438
IFN-γ	9.23 (8.33; 11.98)	11.76 (8.45; 14.32)	0.944	11.31 (9.01; 13.14)	10.11 (9.11; 10.99)	0.086
IL-1RA	47.24 (35.25; 68.65)	52.42 (43.67; 75.81)	0.940	48.99 (38.13; 86.43)	52.20 (38.13; 75.37)	0.651
IL-1α	49.69 (43.25; 63.39)	55.43 (53.38; 63.77)	0.057	56.95 (46.15; 71.46)	54.67 (50.12; 74.52)	0.461
IL-1β	2.34 (2.06; 2.99)	2.56 (2.29; 2.93)	0.907	2.64 (1.85; 2.98)	2.17 (1.52; 2.64)	0.139
IL-2	4.42 (4.23; 4.99)	4.67 (4.30; 5.65)	0.153	5.16 (4.67; 6.11)	5.29 (4.62; 6.11)	0.564
IL-3	3.22 (2.55; 3.43)	3.07 (2.81; 3.69)	0.750	3.32 (2.81; 4.05)	3.25 (2.81; 4.08)	0.457
IL-4	78.37 (69.57; 120.05)	80.70 (76.83; 111.72)	0.509	75.10 (65.15; 100.79)	79.52 (65.74; 89.87)	0.637
IL-5	1.98 (1.34; 3.23)	2.07 (1.40; 2.62)	0.991	1.98 (1.43; 2.53)	1.87 (1.73; 2.49)	0.619
IL-6	5.75 (4.38; 10.25)	5.86 (4.45; 7.12)	0.054	5.47 (4.15; 10.24)	4.89 (3.39; 7.03)	0.041 *
IL-7	10.33 (7.69; 12.92)	10.67 (9.62; 12.06)	0.551	10.33 (7.69; 12.93)	9.57 (7.74; 11.41)	0.632
IL-8	14.75 (9.04; 18.32)	13.63 (10.40; 16.99)	0.100	11.76 (7.56; 22.41)	10.72 (7.34; 17.61)	0.291
IL-9	3.73 (2.63; 3.97)	3.44 (2.54; 3.90)	0.236	3.80 (2.33; 4.61)	2.94 (2.11; 3.84)	0.386
IL-10	9.07 (7.78; 11.34)	9.24 (7.24; 9.91)	0.216	7.89 (6.15; 11.19)	7.39 (6.05; 8.87)	0.169
IL-12p40	42.10 (39.05; 48.45)	44.23 (42.62; 46.19)	0.654	40.66 (33.10; 53.31)	39.46 (26.89; 46.83)	0.050 *
IL-12p70	6.71 (5.69; 7.13)	7.24 (6.43; 9.19)	0.417	6.85 (5.96; 8.89)	7.28 (6.14; 8.59)	0.765
IL-13	14.52 (11.61; 17.91)	16.00 (12.93; 21.11)	0.567	13.52 (10.64; 15.27)	12.97 (10.56; 16.01)	0.411
IL-15	6.47 (5.25; 7.31)	6.70 (5.56; 7.65)	0.687	6.17 (4.84; 7.39)	5.91 (4.97; 7.49)	0.540
IL-17A	4.18 (3.51; 5.24)	4.59 (4.18; 5.15)	0.581	4.43 (3.73; 6.32)	3.97 (3.21; 5.24)	0.172
TNF-α	19.14 (14.61; 25.91)	22.06 (14.77; 28.79)	0.798	16.09 (10.49; 25.48)	16.15 (11.24; 20.52)	0.161
TNF-β	8.29 (6.00; 13.81)	6.98 (6.12; 18.92)	0.569	6.24 (2.89; 13.51)	5.88 (2.50; 8.90)	0.280

IFN—interferon; IL—interleukin; TNF—tumor necrosis factor. *—*p* < 0.05 for the Wilcoxon signed-rank test.

**Table 5:** Serum concentrations of the cytokines (pg/mL) in patients without MetS during 6 weeks of aripiprazole therapy, Me (Q1; Q3).

Indicators	Patients without MetS (n = 11)		
	Before	After	*p*-Value
IFN-α2	17.60 (12.85; 34.04)	22.87 (11.38; 27.33)	0.131
IFN-γ	9.85 (8.03; 14.19)	9.33 (8.33; 12.58)	0.082
IL-1α	55.31 (40.39; 62.64)	54.18 (44.46; 55.67)	0.721
IL-1β	2.26 (1.98; 2.57)	2.01 (1.39; 2.50)	0.333
IL-2	5.40 (3.93; 6.65)	5.49 (4.67; 6.11)	0.929
IL-3	3.34 (2.81; 4.22)	3.58 (2.83; 4.05)	0.721
IL-4	85.19 (70.40; 108.60)	78.46 (72.14; 101.36)	0.477
IL-5	2.41 (2.23; 2.56)	2.18 (1.70; 3.00)	0.594
IL-6	4.83 (3.59; 8.25)	5.16 (3.80; 7.39)	0.965
IL-7	9.88 (8.02; 12.19)	9.50 (8.49; 12.19)	0.610
IL-8	16.58 (7.85; 30.76)	10.03 (7.70; 17.83)	0.859
IL-9	3.04 (2.76; 4.01)	2.89 (2.11; 4.10)	0.575
IL-10	8.00 (5.84; 10.72)	7.83 (5.14; 10.35)	0.131
IL-12p40	40.18 (32.22; 48.98)	38.33 (27.89; 47.52)	0.477
IL-12p70	7.13 (5.53; 9.69)	6.50 (6.06; 7.38)	0.197
IL-13	13.58 (11.33; 17.54)	12.18 (9.84; 15.52)	0.285
IL-15	5.98 (5.38; 7.49)	5.42 (4.84; 7.75)	0.185
IL-17A	4.45 (3.58; 5.03)	4.16 (3.26; 5.15)	0.153
TNF-α	19.13 (9.73; 30.40)	13.89 (10.67; 23.87)	0.333
TNF-β	5.02 (2.69; 6.97)	5.51 (2.36; 7.41)	0.859

IFN—interferon; IL—interleukin; TNF—tumor necrosis factor.

## Data Availability

Authors can confirm that all relevant data are included in the article.
